# Fatigue dataset of high-entropy alloys

**DOI:** 10.1038/s41597-022-01368-5

**Published:** 2022-07-06

**Authors:** Shiyi Chen, Xuesong Fan, Baldur Steingrimsson, Qingang Xiong, Weidong Li, Peter K. Liaw

**Affiliations:** 1 Department of Materials Science and Engineering, The University of Tennessee, Knoxville, TN 37996 USA; 2 Imagars LLC, Hillsboro, OR 97124 USA; 3 State Key Laboratory of Pulp and Paper Engineering, South China University of Technology, Guangzhou, 510640 China

**Keywords:** Metals and alloys, Mechanical properties

## Abstract

Fatigue failure of metallic structures is of great concern to industrial applications. A material will not be practically useful if it is prone to fatigue failures. To take the advantage of lately emerged high-entropy alloys (HEAs) for designing novel fatigue-resistant alloys, we compiled a fatigue database of HEAs from the literature reported until the beginning of 2022. The database is subdivided into three categories, i.e., low-cycle fatigue (LCF), high-cycle fatigue (HCF), and fatigue crack growth rate (FCGR), which contain 15, 23, and 28 distinct data records, respectively. Each data record in any of three categories is characteristic of a summary, which is comprised of alloy compositions, key fatigue properties, and additional information influential to, or interrelated with, fatigue (e.g., material processing history, phase constitution, grain size, uniaxial tensile properties, and fatigue testing conditions), and an individual dataset, which makes up the original fatigue testing curve. Some representative individual datasets in each category are graphically visualized. The dataset is hosted in an open data repository, Materials Cloud.

## Abstract (summary)

**Table:** 

Measurement(s)	Low cycle fatigue • High cycle fatigue • Fatigue crack growth rate
Technology Type(s)	mechanical testing system
Factor Type(s)	Alloy system • Temperature • Processing condition • Phases • Grain size • Stress ratio • Frequency

## Background & Summary

High-entropy alloys (HEAs) are an emerging field of material research. Ever since its first formal reports^1,2^, HEAs have been drawing increasing attention from both the academia and industry and are becoming one of the hottest research areas in materials science^3,4^. These alloys stand out from many other metallic materials because of their unprecedented chemistries (i.e., multi-principal elements), distinctive lattices (i.e., highly hybrid and distorted), unique microstructures (i.e., entropy-stabilized phase structures), and therefore, they exhibit compelling properties (e.g., high strengths derived in part from significant solid-solution strengthening)^5–16^. Consequently, HEAs hold great potential as next-generation high-performing alloys by tailoring their composition-microstructure-property relationships using physical metallurgy principles. Many aspects of HEAs are being investigated concurrently by numerous researchers from all over the world, generating hundreds to thousands of publications each year. To provide an example, close to 800 journal papers were published on HEAs in the calendar year of 2019^3^. Abundant publications inevitably give rise to enormous amounts of data. These data bear unignorable values in distilling insights and patterns with artificial intelligence (AI)^17,18^ when they are compiled and centralized. In fact, researchers have compiled uniaxial tension and compression data of HEAs in plain tables^19,20^ and database formats^21^.

We realize that the fatigue data of HEAs hold the same weight in importance in facilitating the invention and development of next-generation metallic materials for applications across industries. Static uniaxial mechanical performance of a material set its basis for applications. It is fatigue that ultimately dictates the life span of the material in applications. This argument is self-explanatory, given the facts that the majority of load-bearing metallic structures (e.g., those in the automotive and aerospace industries) experiences dynamic cyclic loading, instead of static loading, and approximately 90% of structural failures are caused by fatigue^22–24^. It is, therefore, valuable to have a database of fatigue properties for HEAs in place.

With such thinking, we initiated an initiative to glean all fatigue-related data of HEAs from the literature, compile them in a structured way, and stock them in a publicly-accessible depository. The dataset is largely based upon the pre-existing compilations in two of our recent publications^3,22^, but incorporates new publications since then. In addition, we are furnishing extra information associated with fatigue data, such as the details of testing conditions and corresponding uniaxial tensile data from the same sources. The data are broken down into three categories, namely, low-cycle fatigue (LCF), high-cycle fatigue (HCF), and fatigue-crack-growth rate (FCGR), with each category containing tens of independent records. We hope that our database merely serves as a starting point, from which the fatigue database of HEAs can continuously evolve and grow. We call for contributions from all researchers in the field, especially those studying the fatigue behavior of HEAs, to sustain the continuous growth and maturity of the database.

## Methods

The data are sourced from the publications on fatigue properties of HEAs, since the appearance of the very first publication in 2012, until the beginning of the calendar year of 2022. Throughout this about 9-year span, about 80% fatigue data were published after 2018. Web of Science and Google Scholar were two of the main search engines used for locating the publications in this sub-field. Following downloading of the publications, figures therein containing fatigue data were screenshotted, the data points were digitized with WebPlotDigitizer version 4.5^25^ and stored in an Excel template. Alongside the extracted and recorded data were alloy compositions as well as a number of factors that could influence the fatigue properties of alloys, such as processing history, grain size, uniaxial tensile properties, fatigue testing conditions, etc.

The raw data existing in the literature were originally reported in varied units. For consistency, unit conversions were applied to the data extracted, so that the data in the database had consistent units. On occasion, there were ambiguities in certain data or essential data were missing from the publications. In such instances, we emailed the authors asking for clarifications or providing us with raw data. If the authors did not reply, even after a couple of follow-up attempts, we chose to drop the records, in order to prevent any confusion or misrepresentation.

Additionally, we expanded the fatigue data originally reported in the literature by adding extra data columns that directly derivable from the original data. In LCF, the following relationships exist.

1 $$\frac{\Delta \varepsilon }{2}=\frac{\Delta {\varepsilon }_{el}}{2}+\frac{\Delta {\varepsilon }_{pl}}{2},$$

and,

2 $$\Delta \varepsilon ={\varepsilon }_{max}\left(1-R\right),$$

where $\frac{\Delta \varepsilon }{2}$, $\frac{\Delta {\varepsilon }_{el}}{2}$, $\frac{\Delta {\varepsilon }_{pl}}{2}$ are the total, elastic, and plastic strain amplitudes, Δ*ɛ* is the strain range, *ɛ*_*max*_ is the maximum applied strain, and *R* = *ɛ*_min_/*ɛ*_max_ is the strain ratio. $\frac{\Delta \varepsilon }{2}$, $\frac{\Delta {\varepsilon }_{el}}{2}$, $\frac{\Delta {\varepsilon }_{pl}}{2}$, and *ɛ*_*max*_ are all made available in the database by applying Eqs. (1) and (2) to the reported literature data.

Similarly, the following relationships exist in HCF.

3 $$\frac{\Delta \sigma }{2}=\frac{{\sigma }_{max}}{2}\left(1-R\right),$$

where $\frac{\Delta \sigma }{2}$ is the stress amplitude, *σ*_*max*_ is the maximum applied strain, and $R={\sigma }_{{\rm{\min }}}/{\sigma }_{{\rm{\max }}}$ is the stress ratio. Both $\frac{\Delta \sigma }{2}$ and *σ*_*max*_ are made available in the database by applying Eq. (3) to one of the two quantities reported in the literature.

## Data Records

The data are structured in seven color-coded worksheets of a Microsoft Excel workbook by category, which are “Front page”, “LCF summary”, “LCF individual dataset”, “HCF summary”, “HCF individual dataset”, “FCGR summary”, and “FCGR individual dataset”. The Excel database is archived in the open-access data repository, Materials Cloud (URL: https://www.materialscloud.org/), for ready access by researchers. The full database record in Materials Clouds reads as “Shiyi Chen, Xuesong Fan, Weidong Li, Baldur Steingrimsson, Peter Liaw, *Fatigue database of high entropy alloys*, Materials Cloud Archive **2022.11** (2022), doi: 10.24435/materialscloud:s6–39”^26^.

**Fig. 1:**
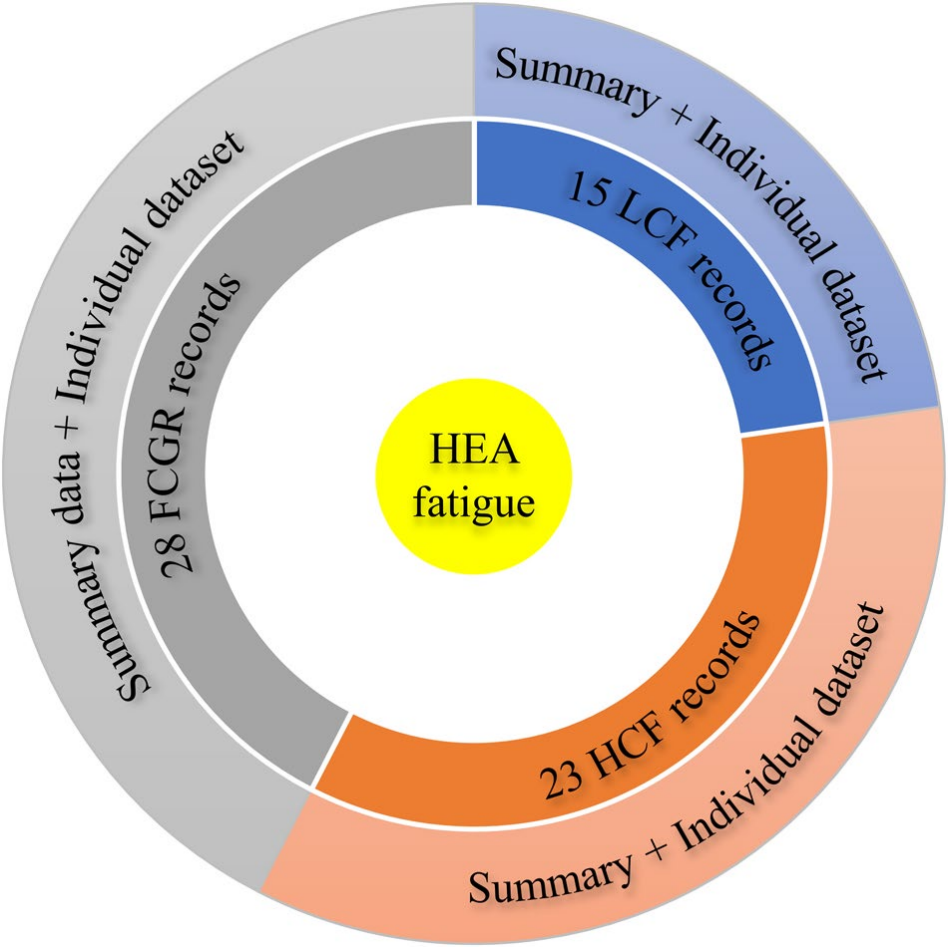
Schematic structure of the high-entropy alloy fatigue database, which is comprised of 15 low-cycle fatigue (LCF), 23 high-cycle fatigue (HCF), and 28 fatigue crack growth rate (FCGR) records, with each record further constituted by an extended summary covering conditions from processing all the way to fatigue testing, and an attached individual fatigue dataset.

The data are subdivided into three broad groups based on the types of fatigue tests conducted, namely, low-cycle fatigue (LCF), high-cycle fatigue (HCF), and fatigue-crack-growth rate (FCGR). LCF, HCF, and FCGR consist of 15, 23, and 28 date records, respectively, as schematically illustrated in Fig. 1. Each record represents a uniquely defined metallurgical condition. For instance, the alloy compositions of two records may be identical, but there are at least one other factor distinguishing them from each other, such as the processing history. For all three groups, each individual record is hierarchical, comprising of (1) a high-level summary summarizing all key information about the alloy and its fatigue properties, and (2) a low-level individual dataset delineating the full fatigue testing trajectory. The summary and the individual dataset are linked by the one-to-one indices.

**Fig. 2:**
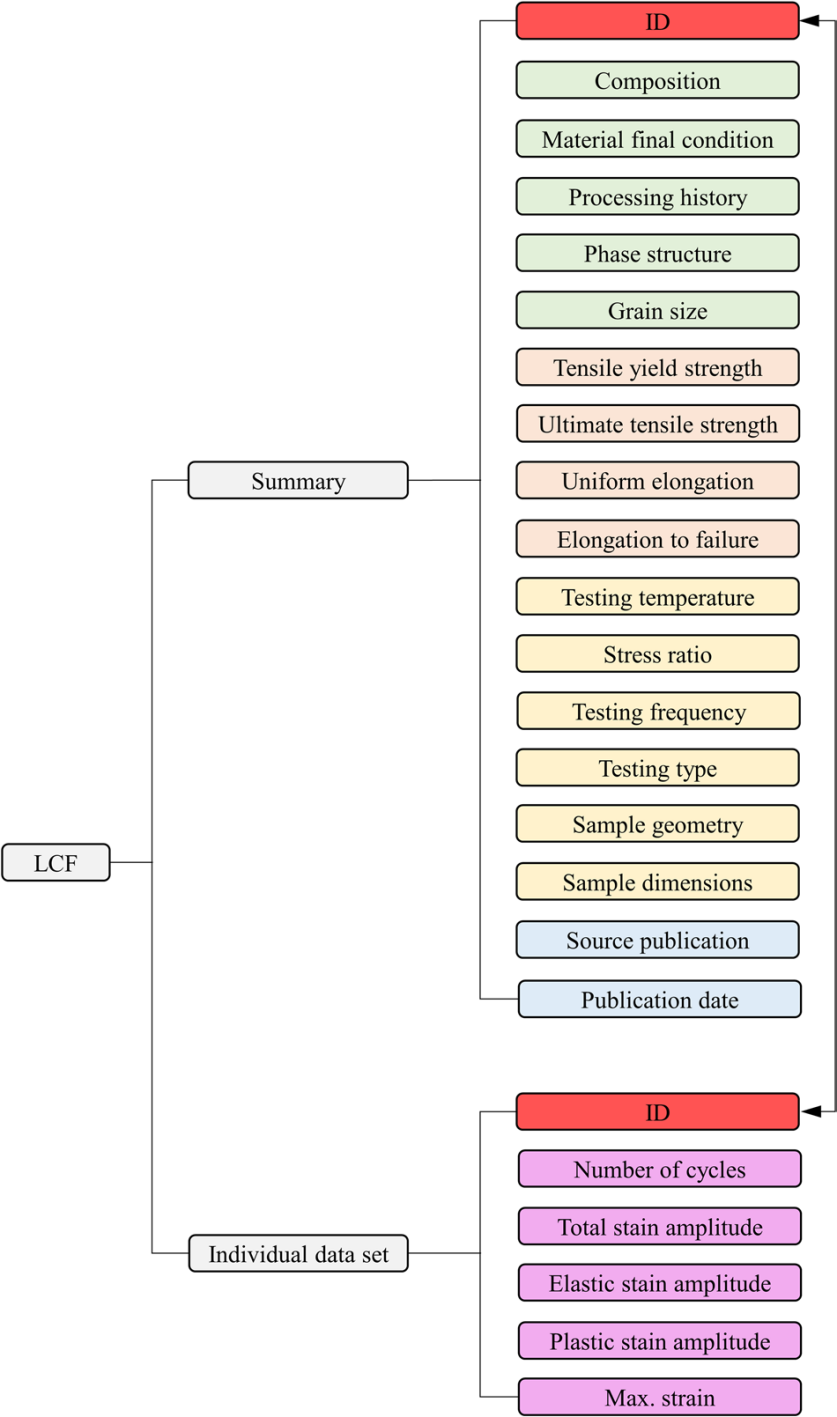
Breakdown of the low-cycle fatigue (LCF) data structure. Summary is categorized by color. Light green: Alloy basics; Light orange: Tensile properties; Light yellow: LCF test; Light blue: Reference.

**Fig. 3:**
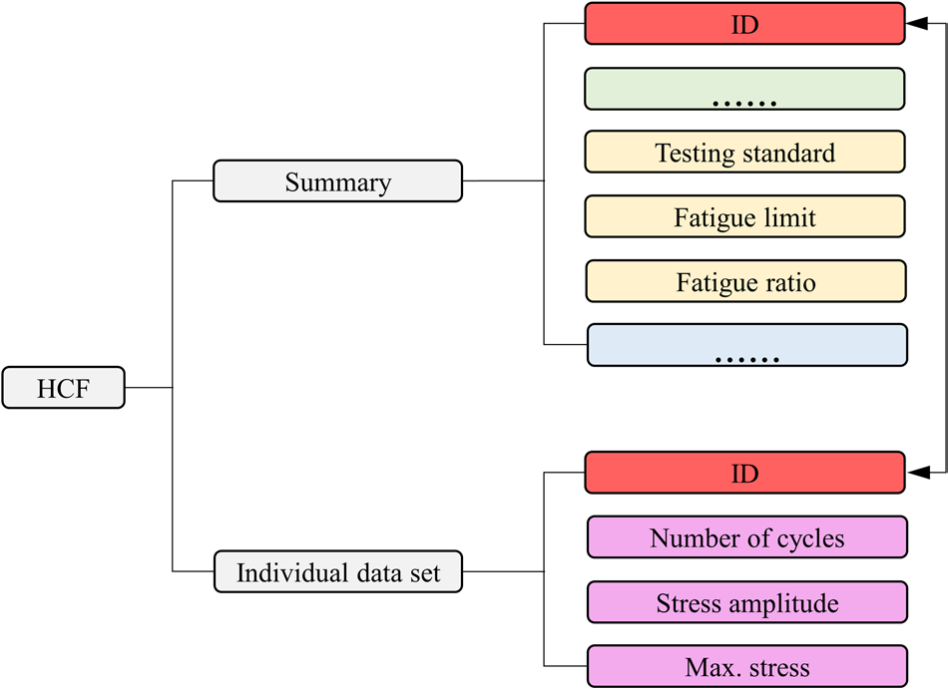
Breakdown of the high-cycle fatigue (HCF) data structure. Most summary data blocks are identical to those found in the LCF data structure and are omitted as indicated by ellipses, and only new blocks are explicitly given.

**Fig. 4:**
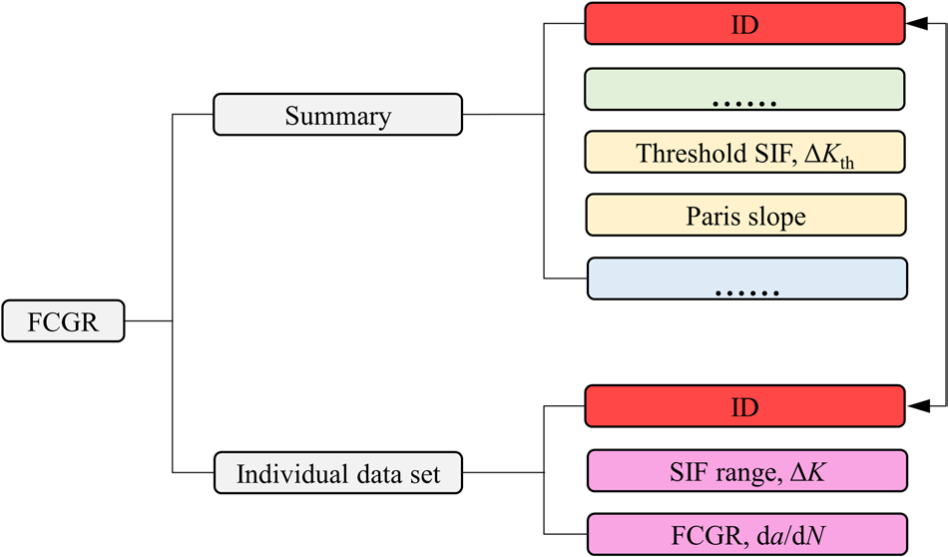
Breakdown of the fatigue-crack-growth-rate (FCGR) data structure. Most summary data blocks are identical to those found in the LCF data structure and are omitted as indicated by ellipses, and only new blocks are explicitly given. SIF is short for stress intensity factor.

The two-level data structure for the LCF data is exemplified in Fig. 2. The summary is further constituted by an array of data that can be broadly classified as the alloy basic information, tensile properties, LCF testing conditions, and the source reference. Each individual dataset is composed of the column data of the number of fatigue cycles, total/elastic/plastic strain amplitude, and maximum strain. HCF and FCGR consist of similar data structures and yet varied fatigue entries in the summary, and completely different columns of data in the individual dataset, as illustrated in Figs. 3 and 4.

## Technical Validation

The original literature data of the same type coming in distinct units are converted to a consistent unit. The accuracy of the extracted data, derived data, and unit conversion is cross-checked and verified multiple times by the team with extensive experiences in HEAs and their fatigue properties.

**Fig. 5:**
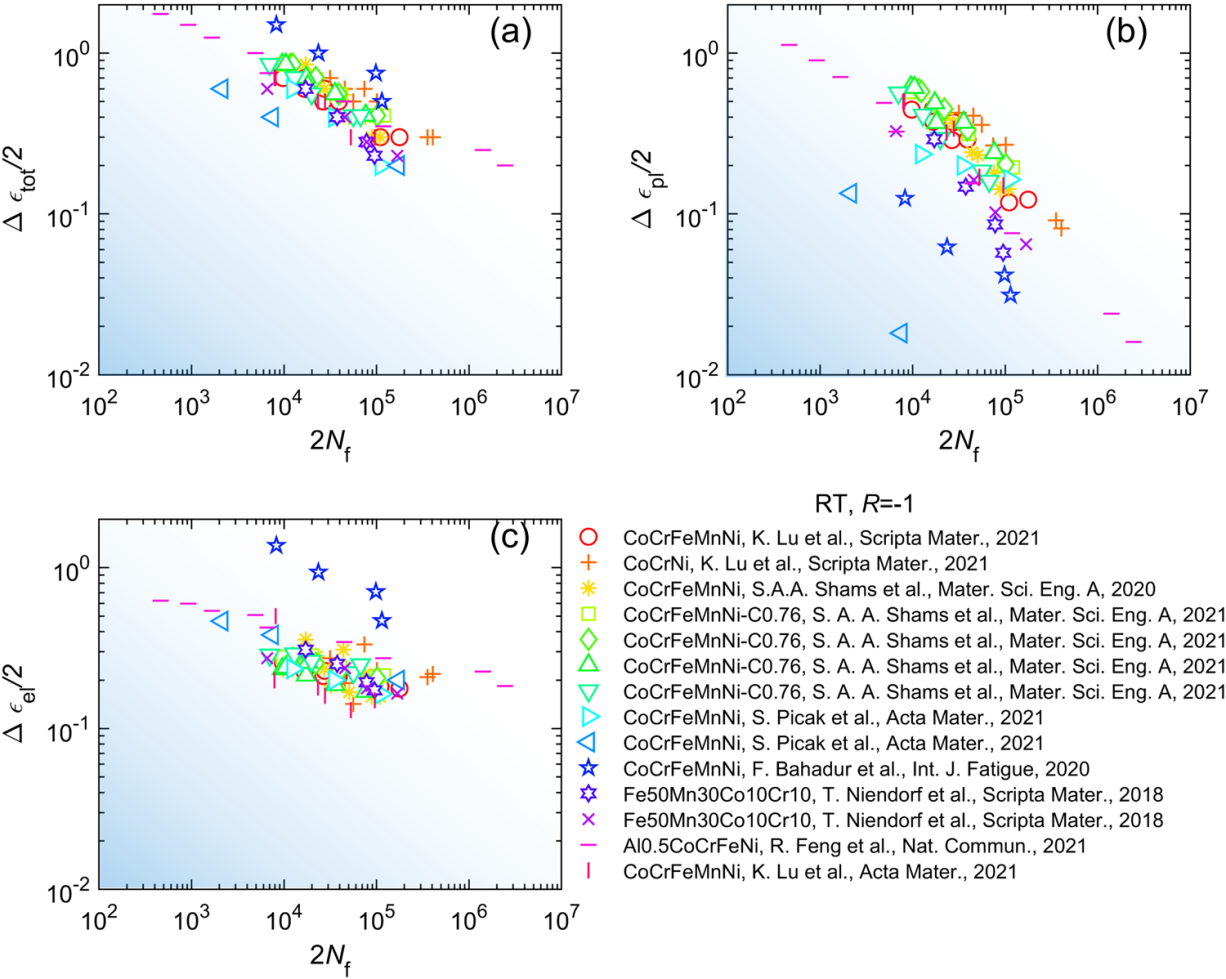
Graphical compilation of low-cycle fatigue data of high-entropy alloys at the temperature of *T* = 298 K and strain ratio of *R* = −1^27–34^. (**a**) Total strain amplitude. (**b**) Plastic strain amplitude. (**c**) Elastic strain amplitude. More data records are found in the database.

**Fig. 6:**
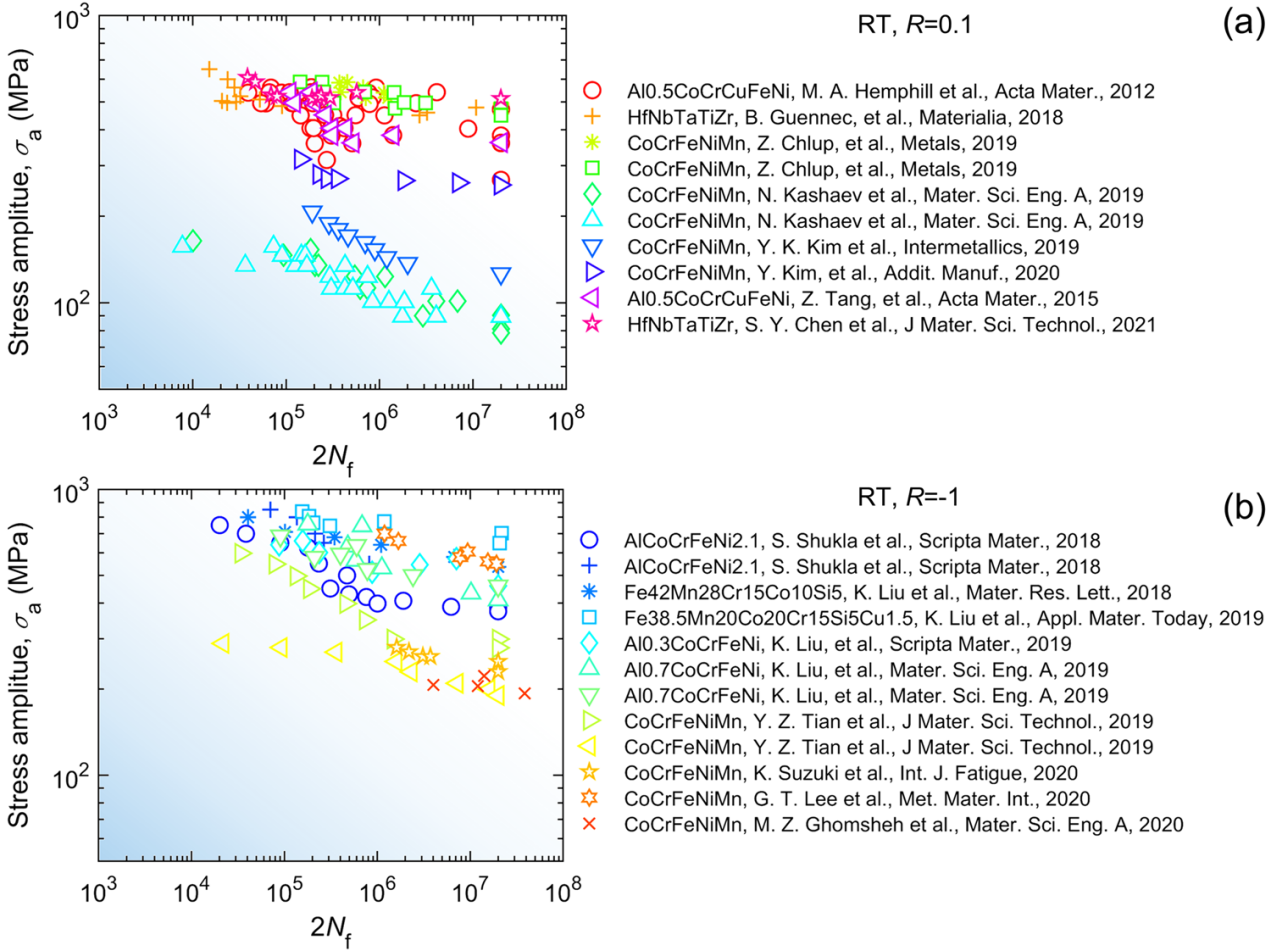
Graphical compilation of high-cycle fatigue data of high-entropy alloys at the temperature of *T* = 298 K and stress ratios of (**a**) *R* = 0.1^35–42^, (**b**) *R* = −1^43–51^. More data records are found in the database.

**Fig. 7:**
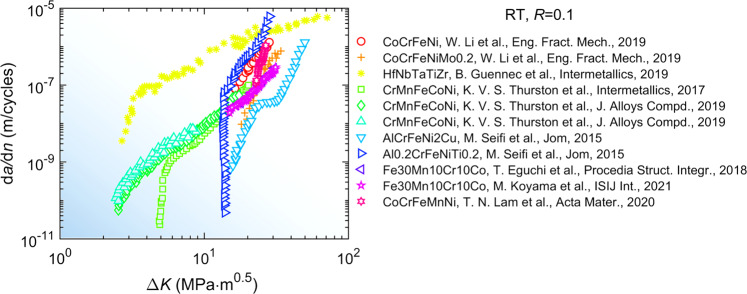
Graphical compilation of fatigue-crack-growth rate data of high-entropy alloys at the temperature of *T* = 298 K and stress ratio of *R* = 0.1. More data records are found in the database^52–57^.

Data visualization serves as an another means of data validation. All records in the individual datasets of LCF, HCF, and FCGR are plotted to visually compare to the source plots in the literature from which the data are extracted. Any spotted discrepancies between our plots and the source ones are investigated and corrected if misrepresentation is confirmed. Some of the plots under the identical temperature and stress or strain ratios are given as follows. Figure 5 depicts the LCF for a variety of alloys at room temperature and the strain ratio of $R={\varepsilon }_{{\rm{\min }}}/{\varepsilon }_{{\rm{\max }}}=-1$ (*ɛ*_min_ and *ɛ*_max_ are the minimum and maximum applied strains), represented by the total, plastic, and elastic strain magnitudes versus number of reversals to failure, 2*N*_f_. Likewise, the selected records in the individual dataset of HCF are visualized in Fig. 6 as stress amplitude, ${\sigma }_{{\rm{a}}}=\left({\sigma }_{{\rm{\max }}}-{\sigma }_{{\rm{\min }}}\right)/2$, as a function of number, 2*N*_f_, for the alloys tested at the stress ratios of *R* = 0.1 and *R* = −1, and at room temperature. The fatigue-crack-growth rate, d*a*/d*n*, versus the stress intensity factor range, Δ*K*, for the selected FCGR records is pictured in Fig. 7. Note that Figs. 5–7 merely represent part of the data from the database. Data under other conditions are not illustrated.

## Usage Notes

The data contained in the database may be used individually or collectively for various purposes. The basic usage may involve comparing the fatigue properties of the HEAs in the database with other materials of interest or with HEAs subsequently tested. As the database continues to grow, the data may be used for AI or machine learning to, for example, facilitate the design of highly fatigue-resistant alloys^17^. Furthermore, many more usage possibilities are waiting to be explored by researchers.

## Data Availability

The code for digitizing and extracting the data from the literature plots is the open-source code WebPlotDigitizer version 4.5^25^, which is freely accessible.
